# A Rare Case of Breast Implant-Associated Anaplastic Large Cell Lymphoma (BIA-ALCL) in a Transgender Patient With Exclusive Smooth Implant Exposure

**DOI:** 10.7759/cureus.115432

**Published:** 2026-08-30

**Authors:** Rocco English, Raul Aguilar, Yin Kan Hwee, Stephanie Greenfield, David Marshall, Karole Oblena, Gary Schwartz

**Affiliations:** 1 Department of Medical Education, Dr. Kiran C. Patel College of Allopathic Medicine, Nova Southeastern University, Fort Lauderdale, USA; 2 Department of Surgery, HCA Florida Northwest Hospital, Margate, USA; 3 Department of Plastic Surgery, Plastic Surgery Specialists of South Florida, Hollywood, USA; 4 Department of Pathology, Memorial Healthcare System, Hollywood, USA

**Keywords:** breast implant-associated anaplastic large cell lymphoma, case report, follicular lymphoma, plastic surgery, smooth breast implants

## Abstract

Breast implant-associated anaplastic large cell lymphoma (BIA-ALCL) is a rare type of T-cell lymphoma that has been strongly associated with textured breast implants. Cases of BIA-ALCL occurring exclusively in patients with smooth breast implant exposure have not been reported. We present the case of a 56-year-old transgender female patient with a history of multiple bilateral breast implant exchanges involving exclusively smooth silicone implants between 2006 and 2023 who presented with progressive unilateral breast swelling and tenderness. Breast imaging demonstrated an enlarged left axillary lymph node without evidence of implant rupture or discrete mass. The patient underwent bilateral implant removal with en bloc total capsulectomy, bilateral pectoralis major repair, and balancing mastopexy. Intraoperatively, a large periprosthetic seroma and thickened left breast capsule were both identified. On further histopathological evaluation, the specimen demonstrated large pleomorphic CD30-positive and anaplastic lymphoma kinase (ALK)-negative lymphoma cells lining the capsule, consistent with BIA-ALCL; this was further confirmed via immunophenotyping and positive T-cell receptor (TCR) gamma gene rearrangement studies. The left breast capsule was also found to have low-grade follicular lymphoma. The contralateral breast demonstrated only reactive histological changes without evidence of malignancy. Ultimately, following complete surgical excision, it was determined that adjuvant therapy was not indicated for this patient. Postoperative follow-up imaging has demonstrated no evidence of disease recurrence thus far. This case challenges current assumptions of BIA-ALCL disease pathogenesis and highlights the need for ongoing investigation into confounding factors associated with the development of this rare form of lymphoma.

## Introduction

Breast implant-associated anaplastic large cell lymphoma (BIA-ALCL) is an uncommon T-cell lymphoma that develops in individuals with breast implants and typically arises from the fibrous capsule surrounding the implant. First reported in 1997, BIA-ALCL was classified by the World Health Organization (WHO) as a distinct type of peripheral T-cell lymphoma in 2016. In 2021, BIA-ALCL was assigned a new diagnostic code within the National Cancer Institute Surveillance, Epidemiology, and End Results Program, allowing the first population-based incidence rate estimate of BIA-ALCL in the United States [1]. The Patient Registry and Outcomes for Breast Implants and Anaplastic Large Cell Lymphoma Etiology and Epidemiology (PROFILE) patient registry tracks BIA-ALCL cases in the United States via voluntary reporting. The most recent PROFILE registry report from 2023 reported that among 330 reported cases of BIA-ALCL, all patients had a history of textured breast implants. Several epidemiologic studies have shown a strong association between textured implants and the development of BIA-ALCL, with estimated lifetime risks varying by manufacturer and implant type [2].

The American Association of Plastic Surgeons reports that a causal relationship has been established between textured breast implants and BIA-ALCL [3]. However, Elameen et al. reported a comparatively similar risk of BIA-ALCL in patients with smooth or textured breast implants in their study [4]. Furthermore, the FDA database for Manufacturer and User Facility Device Experience included 24 cases of BIA-ALCL among 457 BIA-ALCL cases diagnosed in patients who had a smooth implant at the time of diagnosis and a mixed or unknown implant history. Therefore, these reports do not establish an association between BIA-ALCL and smooth implants, particularly given the uncertainty regarding prior textured device exposure [5]. The available evidence has predominantly implicated textured implants in the development of BIA-ALCL. However, in this report, we present a case of BIA-ALCL in a patient with a documented history of exclusively smooth breast implants.

The exact incidence is not known, but patients with BIA-ALCL most often present years after breast implant surgery with delayed breast swelling caused by a peri-implant seroma, although some cases present with a capsular mass or lymphadenopathy. The malignant cells express CD30 and are negative for anaplastic lymphoma kinase (ALK) [6].

When diagnosed at an early stage, treatment consists of implant removal with complete capsulectomy, which is often curative [7]. Systemic treatment, like chemotherapy (i.e., cyclophosphamide, hydroxydaunorubicin, oncovin, and prednisone (CHOP) or brentuximab vedotin) and radiation for unresectable disease, may be warranted when there is extracapsular extension or nodal involvement.

The majority of reported patients diagnosed with BIA-ALCL are cisgender women who underwent breast augmentation or reconstruction following mastectomy. Reports of patients diagnosed with BIA-ALCL involving transgender women are limited, and the coexistence of BIA-ALCL with another lymphoid malignancy is rarely described. To our knowledge, this represents a rare case of a patient diagnosed with BIA-ALCL with a history of only smooth breast implants.

## Case presentation

The patient is a 56-year-old transgender female patient with a chief complaint of swelling of the left breast for a few months. There was no history of immunosuppression or lymphoproliferative disorders. Her past medical history was significant for hypothyroidism managed with levothyroxine; bipolar disorder, treated with sertraline; and hyperlipidemia treated with rosuvastatin. She has a history of multiple breast surgeries. The first breast augmentation with a smooth implant occurred in 2006. She had an implant exchange in 2016 with a smooth round gel 600 cc high-profile (HP) implant, and another implant exchange in 2020, where a 650 cc HP smooth round gel implant was implanted. The last implant exchange occurred in 2023, at which time a 745 cc smooth silicone implant was inserted. She reports a history of swelling of her left breast after the last implant exchange. At that time, an ultrasound revealed a possible ruptured implant, but at the time of surgery, it was reported that a hematoma was found. A previous MRI in November 2023 demonstrated a mass in her axilla which was biopsied and was positive for follicular lymphoma. The MRI was performed out of town and, therefore, was not available to us. Other than a CT scan of the chest, no additional imaging was performed to assess for involvement of other organ systems. After the last exchange, the patient noted swelling in her left breast for several months. Her past medical history is significant for anxiety, bipolar disorder, hypothyroidism, and hyperlipidemia.

Physical examination of the bilateral breasts revealed well-healed inframammary incisions. The left breast was swollen and tender to palpation and had a larger volume than the right breast (Figure 1). The overlying skin was pink, without evidence of necrosis. The bilateral nipples were noninverted and without discharge. There was no evidence of skin dimpling or palpable axillary lymphadenopathy. There were well-healed periareolar scars. 

**Figure 1:**
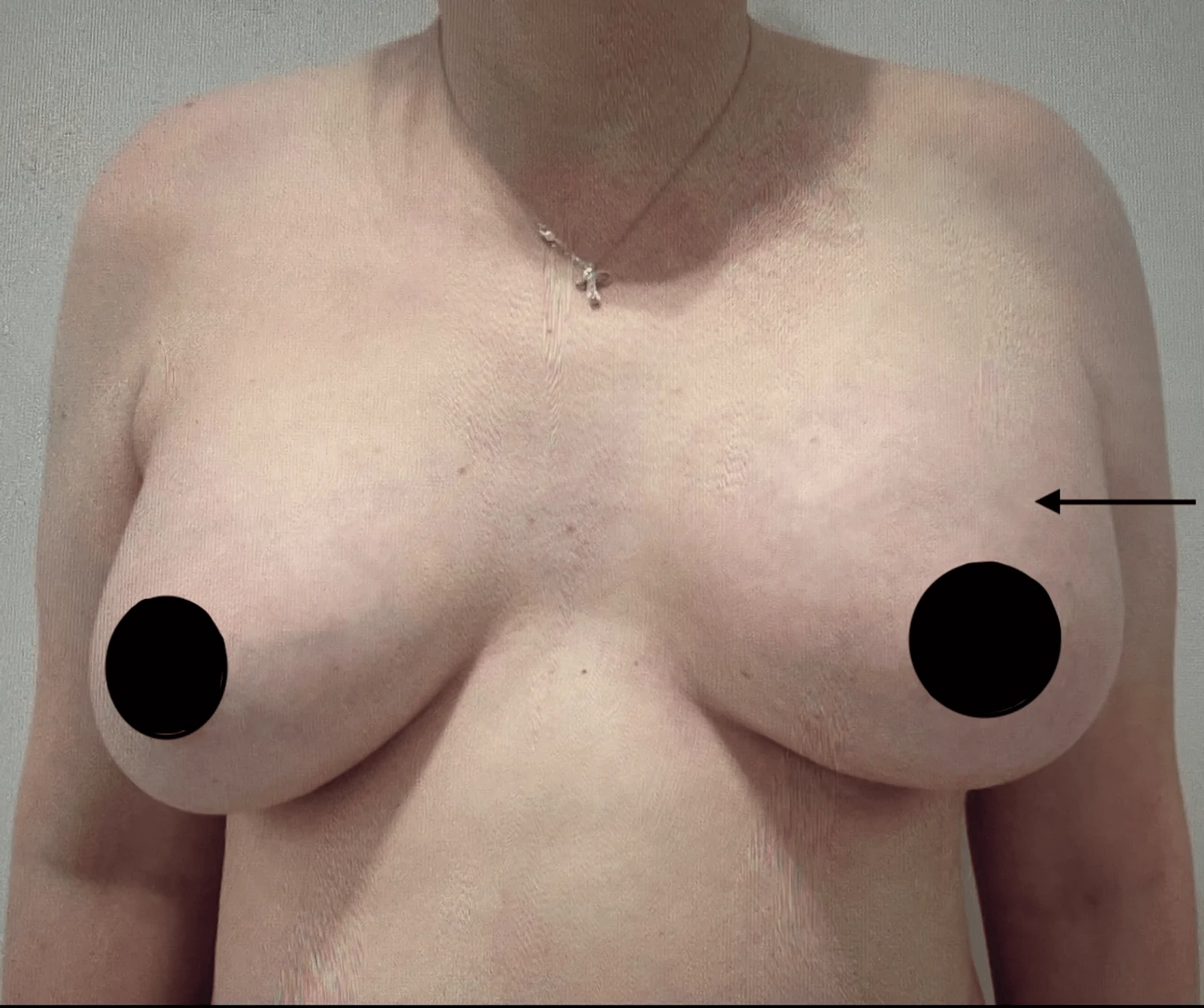
Preoperative photograph just prior to the 2025 surgery demonstrating the swollen left breast (black arrow)

An ultrasound of the left breast was performed to evaluate the swelling. It demonstrated an enlarged left axillary lymph node. There was no evidence of a mass, hematoma, or collection noted. 

The patient was taken to surgery, at which time bilateral implant removal and total capsulectomy were performed, in addition to repair of the bilateral pectoralis major muscles and bilateral balancing mastopexy. The capsule was removed en bloc, and a portion of it was sent as fresh frozen sections for flow cytometry. On frozen section evaluation, there was no evidence of malignancy. Regarding the flow cytometry, a limited antibody panel was performed due to low cellularity. The specimen was predominantly composed of cellular debris/nonhematolymphoid elements. The findings at surgery included intact smooth silicone implants (Figure 2).

**Figure 2:**
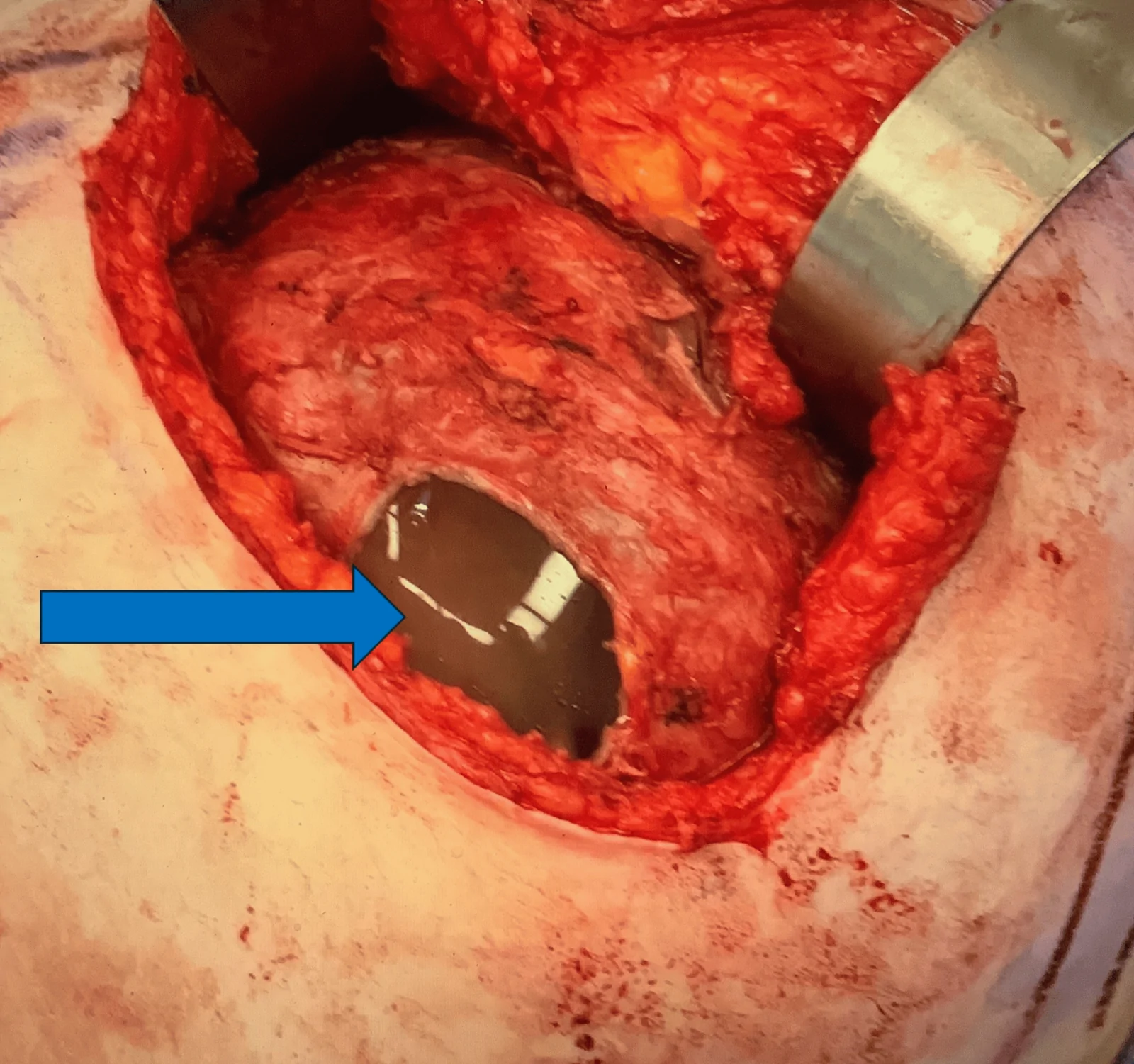
An intraoperative photograph of the left breast implant during the 2025 surgery demonstrating the smooth implant (blue arrow)

The left breast had a thickened capsule associated with periprosthetic fluid (Figure 3).

**Figure 3:**
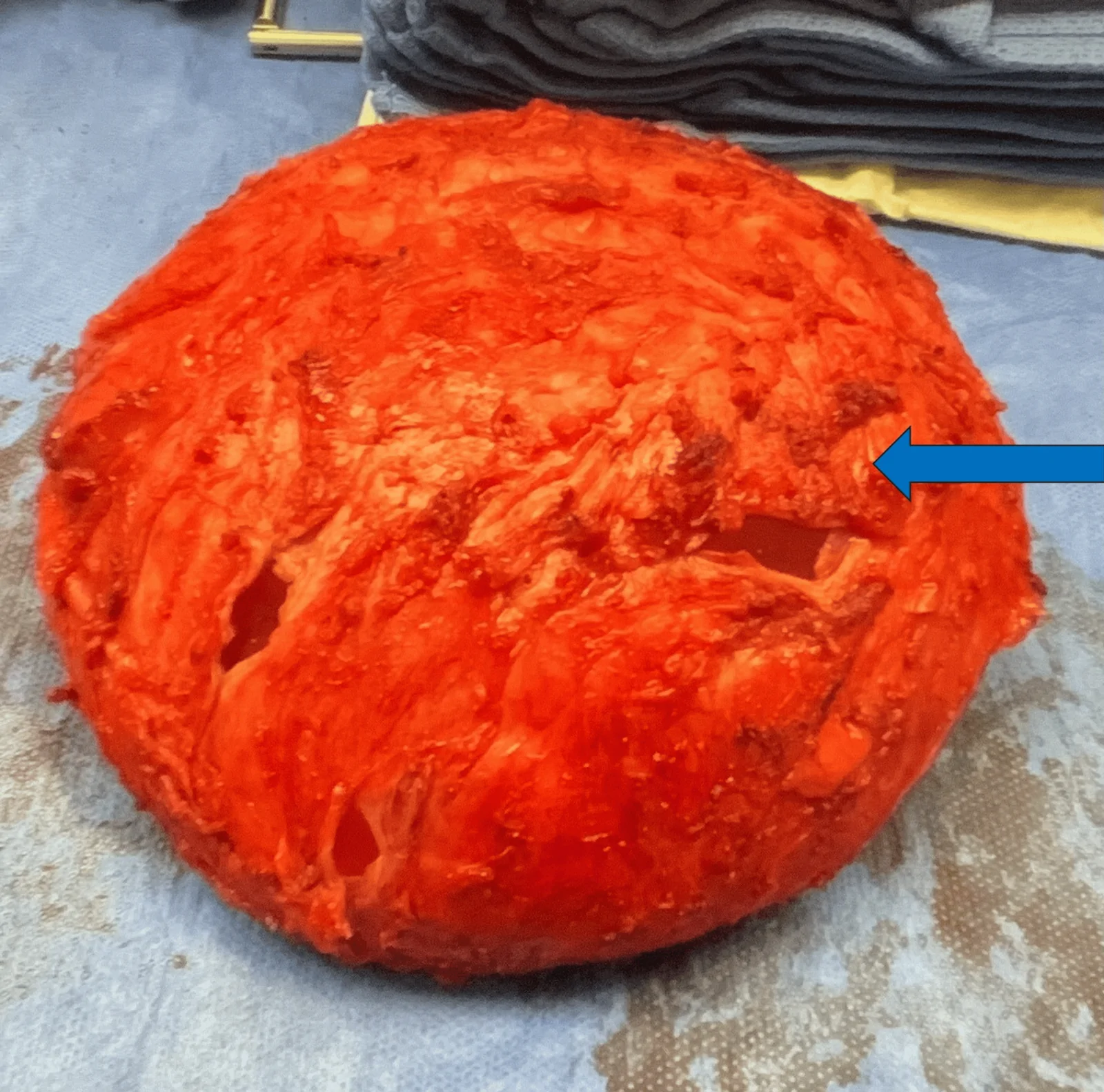
An intraoperative photograph of the left breast implant during the 2025 procedure demonstrating the thick capsule surrounding the left breast implant (blue arrow)

The surgery was performed under general anesthesia in the supine position. The left mastopexy incision was incised. Dissection to the left breast capsule demonstrated thickening of the capsule. There was a large volume of seroma once the capsule was opened. A sample of this fluid as well as a segment of the capsule was sent for fresh frozen evaluation because there was a suspicion of BIA-ALCL. The capsule was completely excised under the pectoralis major muscle and above the chest wall. The pectoralis major muscle was completely detached from the inframammary fold and chest wall. 

On the right breast, the mastopexy skin was de-epithelialized. Dissection down through the breast tissue to the capsule was performed. The capsule was identified and removed en bloc along with the implant (Figure 4).

**Figure 4:**
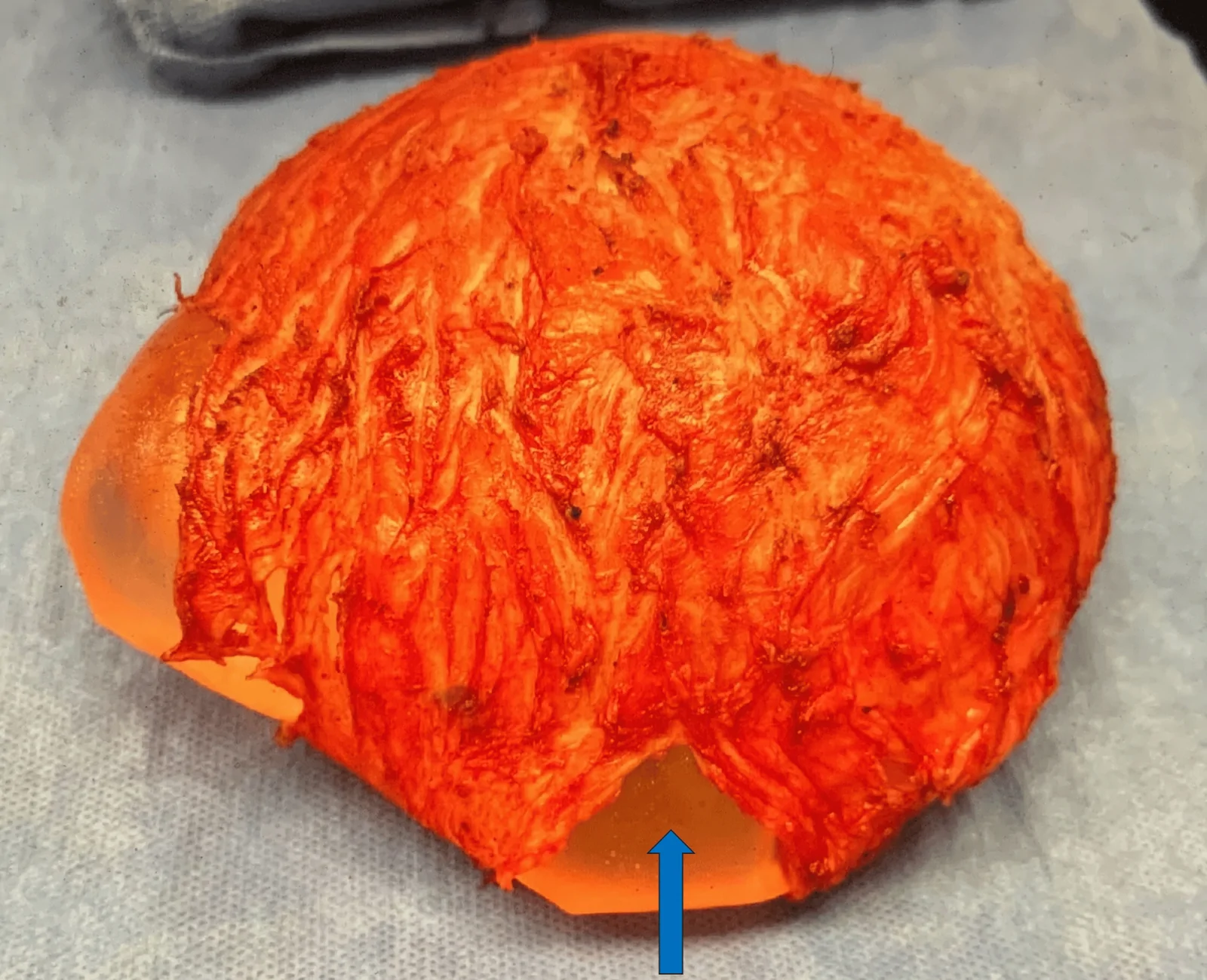
An intraoperative photograph of the right breast implant during the 2025 procedure demonstrating the capsule surrounding the right breast smooth implant (blue arrow)

The sections of the left breast capsule/mastopexy tissue and breast implant showed fibrous tissue with large pleomorphic tumor cells lining the inner surface of the capsule as a distinct layer caught in a fibrinoid and highly necrotic meshwork. Focally, small clusters of tumor cells superficially penetrated the capsule and the fibrous tissue beyond. There was a strong CD30 expression on the tumor cells lining the capsule. In some of the foci, the CD30+ lymphoma cells extended superficially into the underlying fibrous tissue (Figures 5-8).

**Figure 5:**
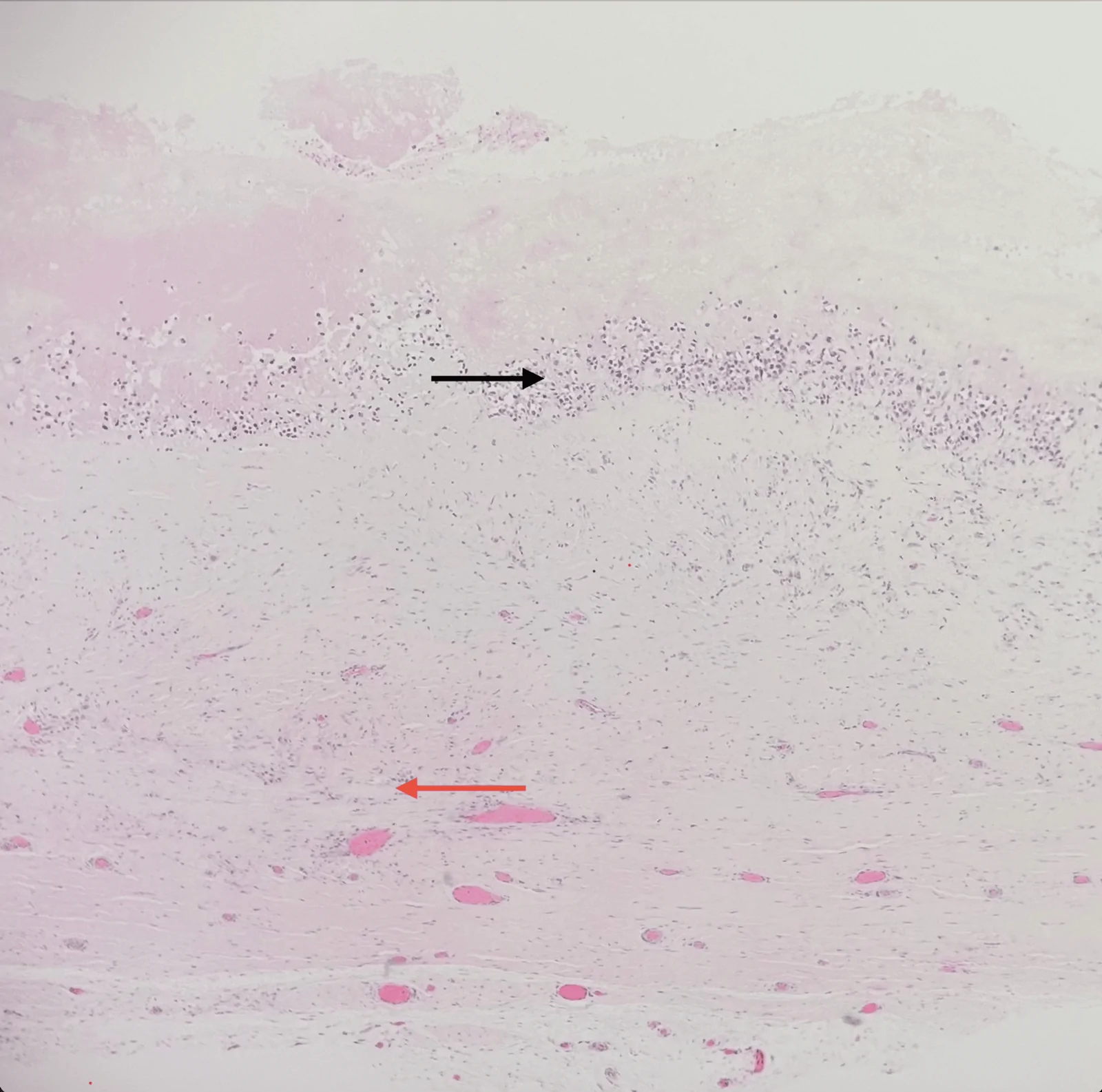
Histopathology of the tumor H&E stain of the tumor at 5x demonstrates the lymphoma cells (black arrows) and the fibrous capsule (red arrows) H&E: hematoxylin and eosin

**Figure 6:**
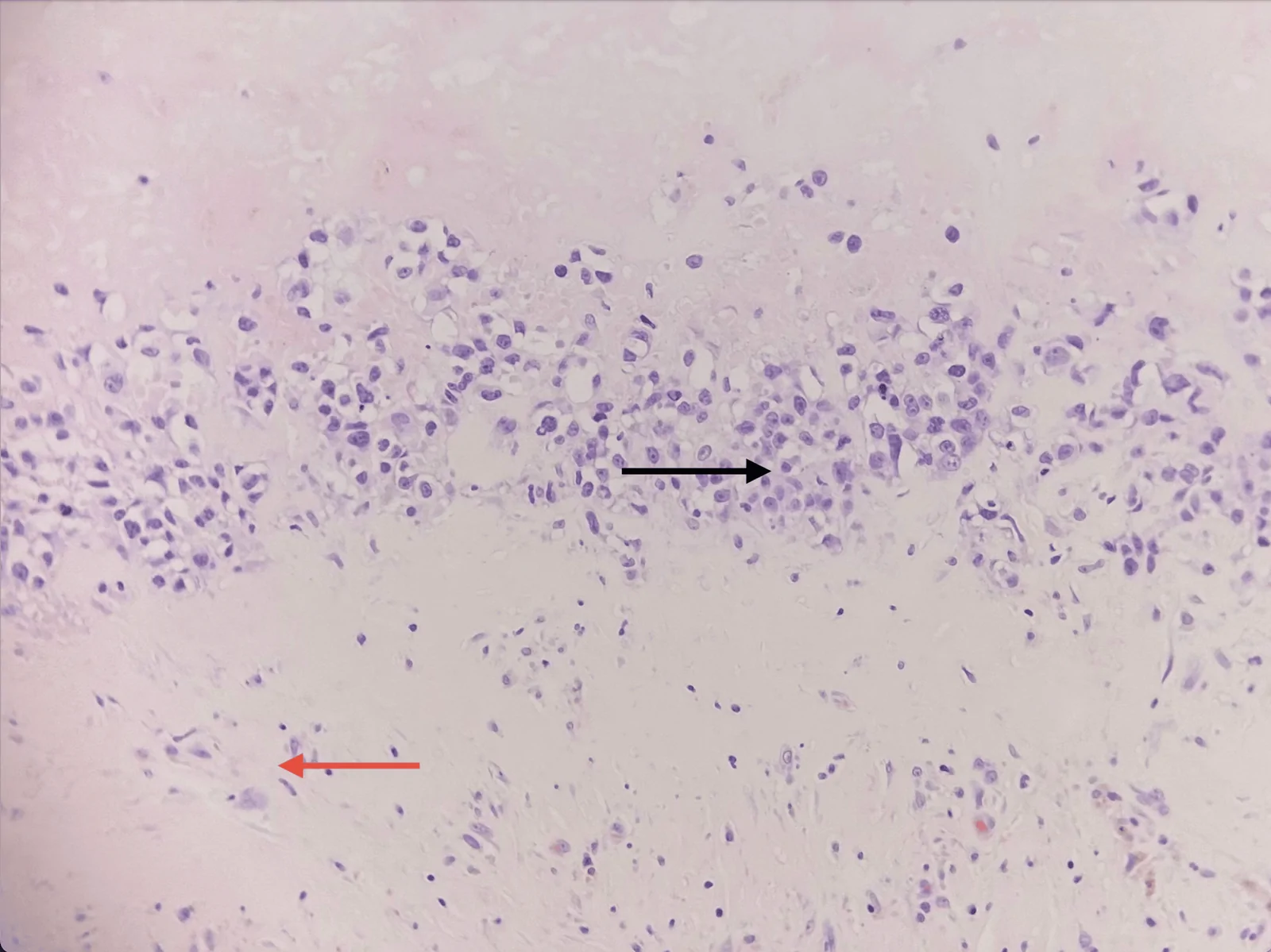
Histopathology of the tumor H&E stain of the tumor at 20x demonstrates the lymphoma cells (black arrows) and the fibrous capsule (red arrows) H&E: hematoxylin and eosin

**Figure 7:**
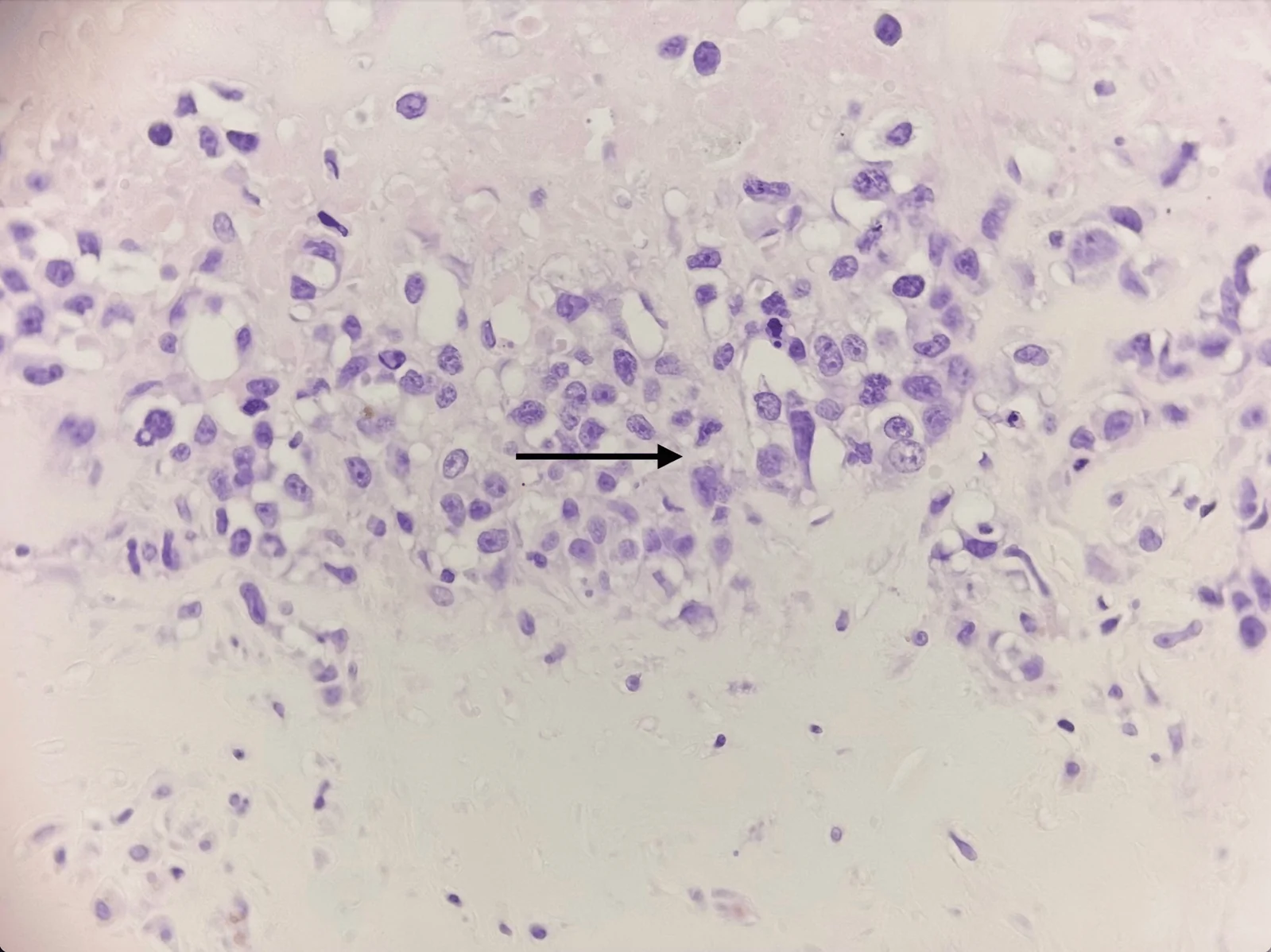
Histopathology of the tumor H&E stain of the tumor at 40x demonstrates the lymphoma cells (black arrows)

**Figure 8:**
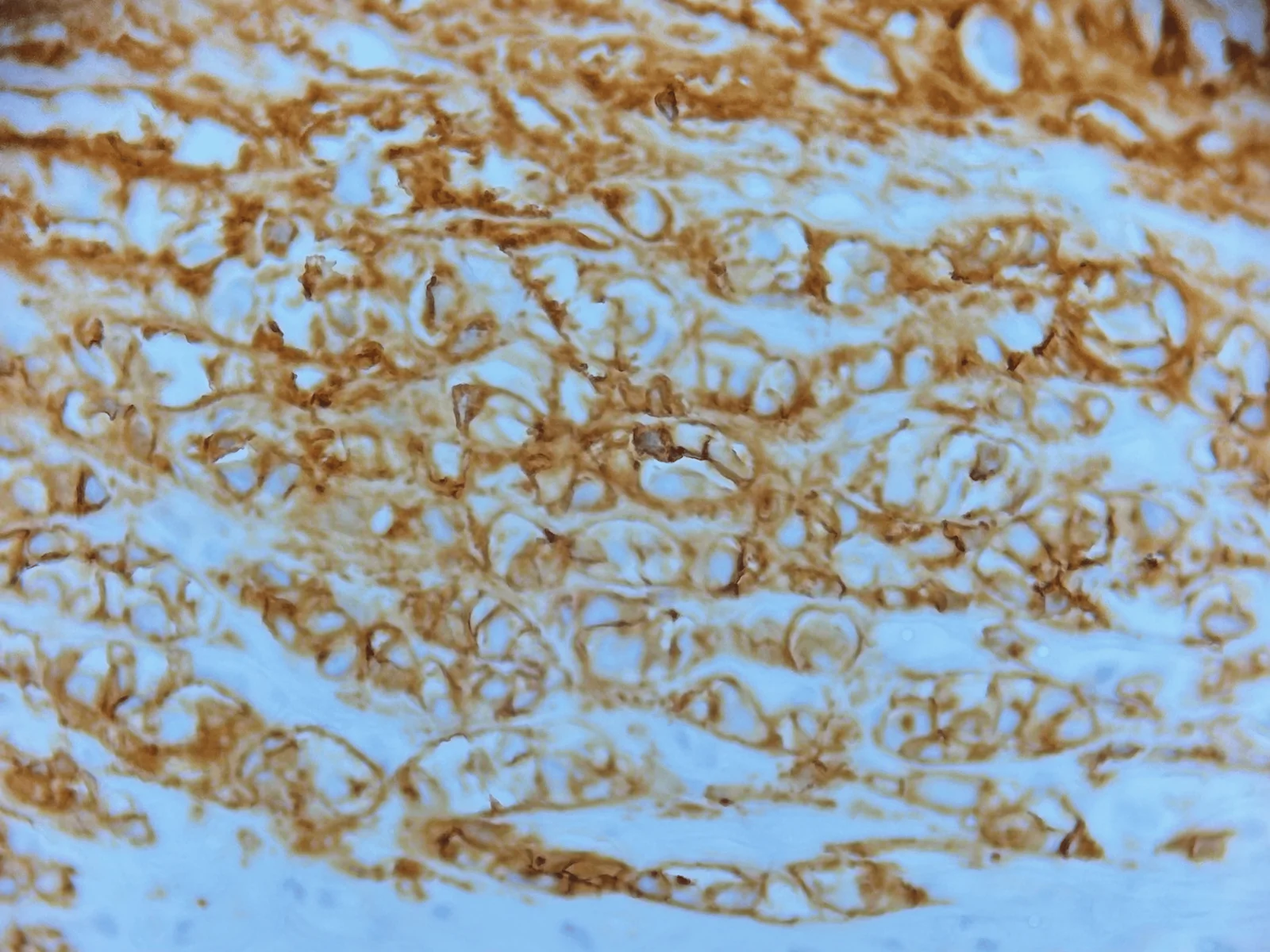
CD30 immunostaining demonstrates the lymphoma cells (D)

The CD30+ lymphoma cells were positive for CD4, CD43, BCL2, GATA3, EMA, MUM1, and Ki-67. The lymphoma cells were partially positive for TIA1, mostly negative for Granzyme with rare positive cells, and negative for CD2 and perforin. The overall morphologic and phenotypic findings were most consistent with an ALK-negative BIA-ALCL. A polymerase chain reaction (PCR) of the T-cell receptor (TCR) gamma gene rearrangement was positive, confirming the diagnosis. Although the majority of the neoplastic cells were mostly confined within the luminal side of the lower necrotic layer without capsular invasion (compatible with a stage T1), there were focal areas where the neoplastic cells showed capsular invasion with associated lymphohistiocytic reaction, corresponding to pathologic stage T3. In addition, there were many small lymphoid aggregates seen at the outer layer of the capsule, which were comprised of small CD20- and PAX5-positive B cells, within CD21-positive follicular dendritic cell meshwork, with positivity for CD10, BCL6, and Bcl-2, consistent with neoplastic lymphoid follicles/low-grade follicular lymphoma. Of note, the patient had a reported prior history of follicular lymphoma on a lymph node biopsy. 

Regarding the right breast implant and capsule, there was fibrous tissue with nonspecific reactive changes. CD30 was negative. There was no morphologic or immunophenotypic support for lymphoma. The timeline and sequence of events are described in Table 1.

**Table 1:** Timeline of events relating to the implant history This table demonstrates the timeline of the breast implant exchange procedures

Date	Event
2006	Bilateral breast augmentation with smooth implants
2016	Bilateral breast implant exchange with Mentor 600 cc HP smooth round gel implants (Mentor Worldwide LLC, Santa Barbara, California)
2020	Bilateral breast implant exchange with Mentor 650 cc HP smooth round gel implants (Mentor Worldwide LLC, Santa Barbara, California)
2023	Bilateral breast implant exchange with 745 cc Allergan soft-touch smooth silicone implants (Allergan Aesthetics (AbbVie), Irvine, California, USA)
2025	Removal of bilateral 745 cc Allergan soft-touch smooth silicone implants (Allergan Aesthetics (AbbVie), Irvine, California, USA) and bilateral balancing mastopexy
2026	Patient discharged from active care

The patient was referred to an oncologist who felt that no adjuvant therapy was required since the entire implant and capsule were removed. A follow-up MRI of the left breast was obtained one year after the last implant was removed. This MRI did not demonstrate any masslike or nonmasslike enhancement. At this point, the patient was discharged from active care.

## Discussion

The evolution of breast implants has reflected decades of surgical innovation aimed at improving aesthetic outcomes, durability, and patient safety. The earliest breast implants were introduced in the 1960s with the creation of modern silicone gel implants, followed by saline-filled implants available as an alternative option. Prior to this, breast augmentation was achieved through a variety of methods including injections of glycerine, autologous fat, ox cartilage, silicone oil, and even snake venom [8]. Initially, modern implants were predominantly smooth-surfaced, but due to concerns of capsular contracture and implant displacement, the development of textured implants began in order to mitigate these complications [9]. The currently available silicone and saline breast implants in the United States have undergone significant design improvements over time. The newer implants reduce inflammation, bacterial adhesion, and biofilm formation, as they have shifted away from textured implants toward smooth and micro-/nanosurface design [10]. Early clinical data on these newer implants show lower rates of complications like capsular contracture and rupture.

Non-Hodgkin lymphoma can present as an aggressive cancer that can involve the breasts, either primary or secondary as a result of advanced systemic disease involvement. While diffuse large B-cell lymphoma is the most common type of lymphoma to be involved with the breasts, the prevalence of T-cell lymphoma is rarely encountered. BIA-ALCL, a subtype of T-cell lymphoma, was first described by Keech and Creech in 1997 [11]. Since then, the number of reported cases has increased, reinforcing a possible association between breast implants and BIA-ALCL. A systematic review published in 2023 by Santanelli di Pompeo et al., including women and female transgender individuals, found the risk for those with textured implants to develop BIA-ALCL to be 1:913 [12]. Patients with BIA-ALCL typically exhibit an indolent clinical course with delayed periprosthetic effusion and breast asymmetry occurring more than one year after implantation (typically 7-9 years) [13]. Regional and distant lymph node involvement can be seen with advanced disease stages. The current National Comprehensive Cancer Network (NCCN) guidelines regarding histologically proven BIA-ALCL include total capsulectomy, excision of associated masses, and biopsy of any suspicious lymph nodes seen on preoperative imaging or intraoperatively. Removal of the contralateral breast implant should be discussed, as 4.6% of reported cases of BIA-ALCL have also involved the contralateral breast [14]. Adjuvant chemotherapy should be considered for patients with extensive disease (stage II-IV on the tumor, node, and metastasis (TNM) staging). Goals of therapy should be individualized with a multidisciplinary team approach involving surgical oncology, hematologic oncology, and plastic surgery teams. Immediate and delayed breast reconstruction may be considered. Although BIA-ALCL is now well-recognized, the exact pathogenesis remains incompletely understood. There is also a lack of data on unique patient populations (transgender individuals), which may limit the generalizability of the current recommendations for management of the disease. It is also important to document if patients have a history of immunodeficiency or lymphoproliferative disorders. 

Our case report details a 56-year-old transgender female patient who underwent multiple bilateral implant exchanges, implant explantation with capsulotomy, bilateral pectoralis major repair, and balancing mastopexy on her most recent surgery for persistent unilateral breast swelling. Final histopathologic evaluation of the left breast capsule/mastopexy specimen demonstrated BIA-ALCL, predominantly with a pathologic stage of pT1 with focal areas of pT3 disease, as well as separate low-grade follicular lymphoma involvement within the left breast capsule. The diagnosis was further supported by immunophenotyping and positive TCR gamma receptor gene rearrangement studies. The right breast capsule showed nonspecific reactive changes without evidence of lymphoma on histopathological evaluation. Following complete en bloc removal of the bilateral implants and capsules, the patient was evaluated by oncology, for which adjuvant therapy was not recommended. This case adds to the small number of reports describing BIA-ALCL in patients with a history of smooth implants and challenges the assumption that the disease occurs exclusively in those with exposure to textured implants. The patient’s history of multiple implant exchanges highlights the potential importance of cumulative exposure and implant history. The presentation with a delayed seroma and capsular thickening mirrors more classic cases and highlights the importance of maintaining suspicion for BIA-ALCL, even when typical risk factors are not present and when the implant history is reportedly smooth-only. The coexistence of low-grade follicular lymphoma within the capsule raises the possibility of a shared local immune or microenvironmental process, although the significance of this finding remains uncertain. Finally, this case contributes to the limited data on BIA-ALCL in transgender patients and underscores the need for broader representation to better inform risk assessment and management of patients.

Teaching point

Although textured implants were originally introduced to address complications associated with smooth implants (i.e., capsular contracture and implant malposition), the almost exclusively linked association between textured implants and BIA-ALCL introduced new safety concerns within the plastic surgery community. In this context, our case report is especially noteworthy because it highlights the first reported case of a patient’s postoperative course complicated by BIA-ALCL with exclusively smooth breast implant exposure, suggesting either an incomplete understanding of BIA-ALCL pathogenesis or the possibility of additional confounding risk factors not yet identified.

## Conclusions

Ultimately, this case highlights a rare presentation of BIA-ALCL in a middle-aged transgender patient with a documented history of multiple smooth breast implant use accompanied by concurrent low-grade follicular lymphoma involving the capsule on final pathology. These findings add to the existing literature and suggest that BIA-ALCL may, in rare circumstances, occur in patients without a history of textured implant exposure. Broader reporting of cases similar to this will be essential for expanding our understanding of BIA-ALCL’s pathogenesis, allowing us to refine our current clinical risk-assessment models in the development of this rare form of lymphoma and further solidify evidence-based management guidelines for future cases.
